# Influence of the cultivation medium and pH on the pigmentation of *Trichophyton rubrum*

**DOI:** 10.1371/journal.pone.0222333

**Published:** 2019-09-10

**Authors:** Oliver Blechert, Hailin Zheng, Xiaohui Zang, Qiong Wang, Weida Liu

**Affiliations:** 1 Department of Medical Mycology, Institute of Dermatology, Chinese Academy of Medical Science and Peking Union Medical College, Nanjing, Jiangsu, People’s Republic of China; 2 Jiangsu Key Laboratory of Molecular Biology for Skin Diseases and STIs, Nanjing, Jiangsu, People's Republic of China; 3 Center for Global Health, School of Public Health, Nanjing Medical University, Nanjing, Jiangsu, People’s Republic of China; Louisiana State University, UNITED STATES

## Abstract

*Trichophyton rubrum* is a human pathogenic fungus. As a dermatophyte it causes athlete's foot, fungal infection of nails, jock itch and ringworm. The pigmentation of *T*. *rubrum* is variable and can range from white or yellow to wine-red. We demonstrate that the pigmentation is strongly influenced by pH. Under alkaline conditions, *T*. *rubrum* has a red pigmentation, whereas at acid conditions, *T*. *rubrum* has a yellow pigmentation. Moreover, the color change immediately from yellow to red by adding NaOH and reverse immediately from red to yellow by adding HCl. We suggest that the chemical compound Xanthomegnin is responsible for red as well for yellow pigmentation in *T*. *rubrum*. To figure out, why *T*. *rubrum* has red pigmentation on Trichophyton medium, adjust to alkaline, but not on Synthetic-Complete medium, also adjusted to alkaline, we measure the pH of liquid media, adjusted to pH 3.5, 6 and 8, over a period of four weeks. The pH of both cultivation media changes significantly, with a maximum of five pH levels. Whereas the Trichophyton medium, initially adjusted to pH 8, stays alkaline, the pH of the Synthetic-Complete medium drops to acid conditions. The acidification of the SC medium and the alkalization of the Trichophyton medium explains the different pigment color of the *T*. *rubrum* colonies.

## Introduction

*Trichophyton rubrum* (Castellani) Sabouraud 1911 is a human pathogenic fungus. As a dermatophyte it causes athlete's foot, fungal infection of nails, jock itch and ringworm. To find an appropriate treatment for the disease, an accurate diagnosis is mandatory. Therefore the diagnosis of the fungal infections involves in many cases microscopic examination and fungal cultivation. According to a survey among dermatologists, direct microscopy was the most important diagnostic tools followed by cultivation of the pathogen [1]. Whereas direct microscopy is often sufficient to detect a fungal infection, the identification of the pathogen requires in most cases fungal cultivation. And a correct identification is important, since the treatment approach is dependent on the pathogen [2]. The appearance of *T*. *rubrum* is to a certain degree inconsistent and the discrimination from related species is sometimes intricate. Variations in the colony surface, culture pigmentation and conidia production are known [3]. Three factors account principal for variations: the genome, the epigenetics and the environment. Since all of these factors are complex, it is challenging to determine which of the factors are responsible for an observed variation. Moreover, a phenotype can be influenced by all three factors.

The pigmentation of *T*. *rubrum* is one such variable trait. The cultivation-media has a great influence on the pigmentation of the fungus. On Sabouraud-Dextrose Agar a weak pigmentation became visible after four weeks of cultivation, whereas on Corn-Meal Dextrose Agar the pigmentation was notably stronger. On Lab-lemco Dextrose Agar the pigmentation was visible already after two weeks and after four weeks the pigmentation was more distinctive in comparison to SDA and CDA media. On all three media, tryptose inhibited the red pigmentation [4].

Beside the environmental influence, it is also reported that strains behaved differently after long-lasting cultivation on artificial medium. [5] isolated 207 *T*. *rubrum* strains and compared the original phenotype with the phenotype after one year cultivation on Sabouraud Agar. Of these strains, 54 showed morphological variations after one year. Some of the strains failed to develop pigmentation until repeated sub-cultivations, other lost pigmentation in the sub-cultures and some even change color from red to yellow or reverse in the different sub-cultures. Despite the fact that the red pigmentation is characteristic for *T*. *rubrum* and the scientific name derived from the colorations, it is a variable trait. The aim of this study was to evaluate which environmental parameters influence the pigmentation of *T*. *rubrum*.

## Materials and methods

### Fungal strains

In all experiments, we used the *T*. *rubrum* strains STRB008 and STRB012 (Table 1). In the test with 46 strains we used additional clinical isolates from Lanzhou University Second Hospital (total 7 strains), Huashan Hospital Fudan University in Shanghai (8), Institute of Dermatology and Hospital for Skin Diseases in Nanjing, Chinese Academy of Medical Sciences & Peking Union Medical Collage (10), Dalian Hospital for Skin Disease (10), Sun Yat-Sen Menmorial Hospital Sun Yat-Sen University in Guangzhou (8) and the strain *T*. *rubrum* CBS 139224 [6]. The clinical strains were isolated for diagnostic purposes and were identified by the clinical personal of the corresponding hospitals. The identifications had been verified by morphological and physiological analysis. Currently the genomes of these strains are sequenced by NGS in the Whole Genome Diversity project and publicly available at the NCBI with the numbers SRX5814810-SRX5814857.

**Table 1:** Strains used in this study.

strain	origin	reference
STRB008	Nanjing	this study
STRB012	Nanjing	this study
TI*	China	Zhan et al. 2018
192	Lanzhou	SRX5814843**
193	Lanzhou	SRX5814842**
194	Lanzhou	SRX5814845**
197	Lanzhou	SRX5814844**
198	Lanzhou	SRX5814847**
200	Lanzhou	SRX5814848**
201	Lanzhou	SRX5814846**
465	Shanghai	SRX5814852**
467	Shanghai	SRX5814853**
480	Shanghai	SRX5814828**
486	Shanghai	SRX5814856**
487	Shanghai	SRX5814854**
503	Shanghai	SRX5814857**
510	Shanghai	SRX5814840**
513	Shanghai	SRX5814829**
774	Nanjing	SRX5814851**
784	Nanjing	SRX5814850**
785	Nanjing	SRX5814822**
786	Nanjing	SRX5814823**
804	Nanjing	SRX5814820**
805	Nanjing	SRX5814821**
806	Nanjing	SRX5814826**
807	Nanjing	SRX5814827**
823	Nanjing	SRX5814824**
852	Nanjing	SRX5814825**
1045	Dalian	SRX5814833**
1046	Dalian	SRX5814832**
1047	Dalian	SRX5814839**
1048	Dalian	SRX5814830**
1049	Dalian	SRX5814837**
1050	Dalian	SRX5814834**
1051	Dalian	SRX5814831**
1052	Dalian	SRX5814838**
1053	Dalian	SRX5814836**
1054	Dalian	SRX5814835**
1055	Guangzhou	SRX5814818**
1056	Guangzhou	SRX5814815**
1057	Guangzhou	SRX5814810**
1058	Guangzhou	SRX5814813**
1059	Guangzhou	SRX5814819**
1060	Guangzhou	SRX5814814**
1063	Guangzhou	SRX5814816**
1096	Guangzhou	SRX5814812**

*CMCC(F)T 1i (CBS 139224)

** T. *rubrum* whole genome diversity project of the Nanjing Hospital for Skin Disease. Sequence Read Archive (SRA) number of NCBI.

### Cultivation media

In the experiments, the Trichophyton medium Nr.1 (Tr1 medium: 40g dextrose, 2.5g casamino acids, 1.8g KH_2_PO_4_, 0.1g MgSO_4_, 18g agar) and Synthetic complete medium (SC medium: 20g glucose, 6.7g bacto-yeast nitrogen base with ammonium sulfate, 2g amino acid mix, 18g agar) were used. For maintenance of the fungus, the pH of the media was adjusted before autoclaving to pH 5.8. In the experiments, the pH was adjusted, after autoclaving, with NaOH respectively HCl. The pH of the agar plates was adjusted to 3.5, 6 and 8.5. The liquid media was adjusted to pH 3.5, 6 and 8. The pH of solid media was measured with pH paper, the pH of liquid media was measured with a pH electrode.

### Cultivation

The fungi were cultivated at 28°C. For the liquid cultures, 250 ml Erlenmeyer flasks were filled with 150 ml medium and incubated with shaking at 120 rpm. For the inoculations the fungi were precultured for 10 days in liquid medium and approximately 0.05g, semi-dry weight, of the precultured mycelium were used as inoculum.

## Results

### Cultivation of *T*. *rubrum* on two different media

We cultivated the strain *T*. *rubrum* STRB012 on two different media and observed two distinct phenotypes. On Tr1 medium (pH adjusted to 5.8) the strain developed a wine-red pigmentation and on SC medium (pH 5.8) a yellow pigmentation. To find out the reason for the differences in the phenotypes, we evaluated the influence of the pH on the pigmentation.

### Cultivation on petri dishes, split in acid and alkaline sections

**Fig 1:**
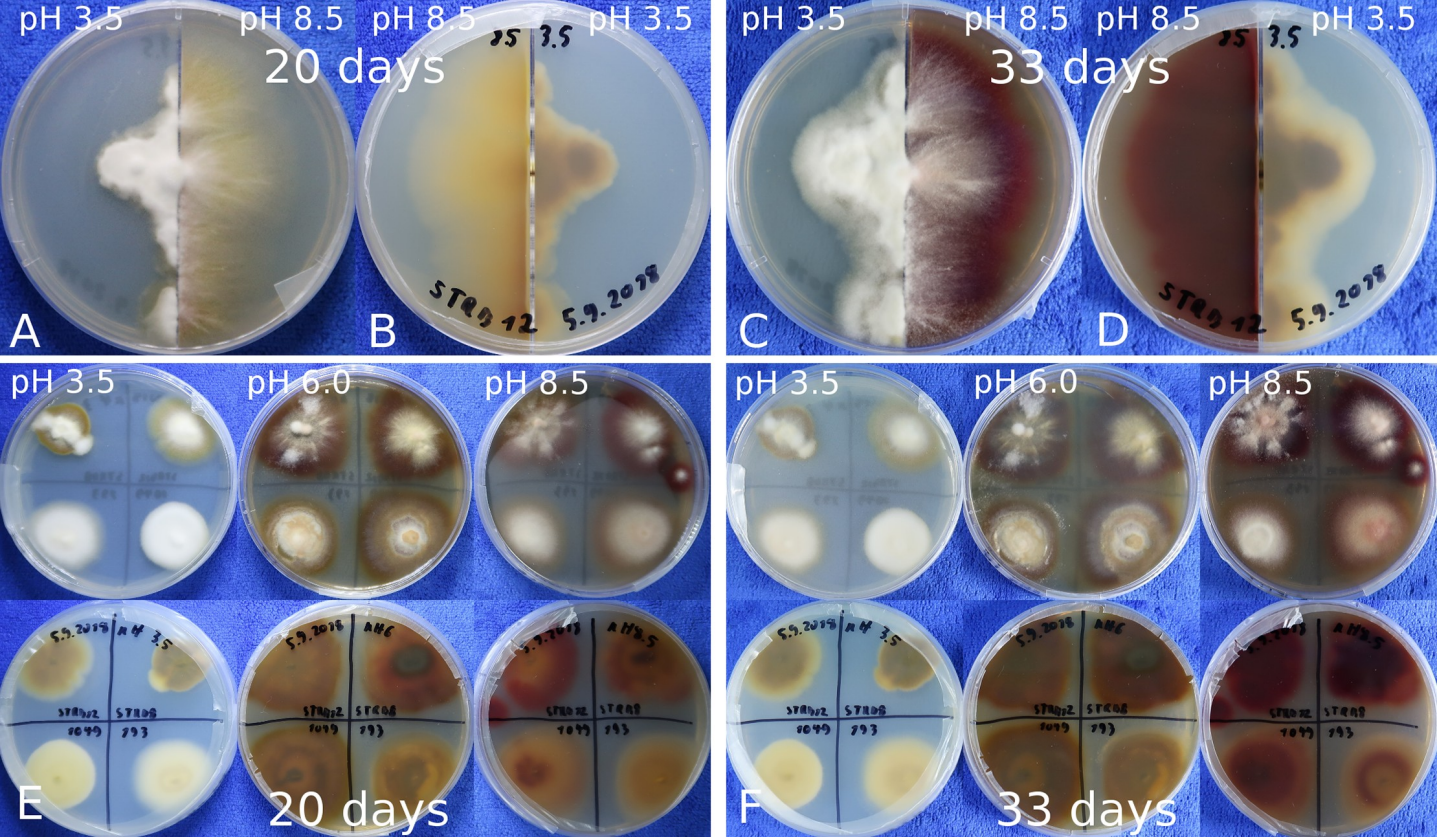
The color development of *T*. *rubrum* on Tr1 media depends on the pH. A-D: *T*. *rubrum* strain STRB012 grown on Tr1 medium in a 9 cm split plate. On right side of the plate the pH was adjusted to 3.5 and on the left side to 8.5 A, B: 20 day after inoculation a yellow to beige pigmentation is visible. C, D: 33 days after inoculation. At high pH a strong wine-red pigmentation is visible, whereas on low pH a brownish to beige pigmentation is visible. E-F: The four *T*. *rubrum* strains STRB008, STRB012, 193 and 1049 were inoculated on Tr1 medium with pH adjusted to 3.5 (left plate), 6 (middle) and 8.5 (right). E: 20 days after inoculation the first strains have a wine-pigmentation. F: 33 days after inoculation all strains have a wine-red pigmentation at high pH, whereas all strains stay whitish to beige under acid conditions.

We chose six *T*. *rubrum* strains (STRB008, STRB012, 197, 804, 1049, 1058) for inoculation on split petri dishes, with a diameter of 9 cm. The pH of the Tr1 medium was adjusted to 3.5 on one side and to 8.5 to the other side (Fig 1A–1D). Whereas the growth on low pH was compact, the growth on alkaline medium was more diffuse. In the first two weeks, the mycelium had a whitish color. Afterwards, the mycelium and culture medium started to get colored. After three weeks, the colonies became yellow brownish, whereas on acid conditions the brownish color was more pronounced. After four weeks on acid conditions, the brownish color got stronger, whereas on alkaline conditions the colony had turned wine-red. We inoculated at total six different *T*. *rubrum* strains on the split petri dishes and all of them had a similar appearance in respect to growth pattern, color expression and time. Under acid conditions the brownish color of two strains on acid were less pronounced in comparison to the other four strains.

### Cultivation on Tr1 and SC medium pH 3.5, 6 and 8.5

Next, aiming to evaluate the variability of the phenotypes among different *T*. *rubrum* strains, we chose the strains STRB008, STRB012, T1 and additional 43 strains of the *T*. *rubrum* Whole Genome project.

We prepared petri dishes with Tr1 and SC medium with a pH of 3.5, 6 and 8.5. On each dish we inoculated four strains. After four weeks the differences in the phenotypes among the different conditions were striking, whereas there was no significant difference among the strains. All strains, cultivated on Tr1 medium (with a pH adjusted to 8.5), developed the wine-red color. On Tr1 medium with a pH of 6, the red color was less pronounced and had a tendency towards beige (Fig 1E and 1F). At low pH as well as on SC medium, none of the strains developed a red pigmentation (Table 2). Comparing the phenotype of the strains at constant conditions, only low differences in the color strength and low differences in the time course were seen.

**Table 2:** Overview of the pigment color of the *T*. *rubrum* grown on two different media for four weeks.

	Tr1 medium	SC medium
pH 3.5*	white to yellowish	white to yellowish brown
pH 6*	dark beige to wine-red	white, yellowish with brown center
pH 8.5*	wine-red	yellow brown with darker center

*initial pH at time of the inoculation

### Reversible color change through HCl respectively NaOH

**Fig 2:**
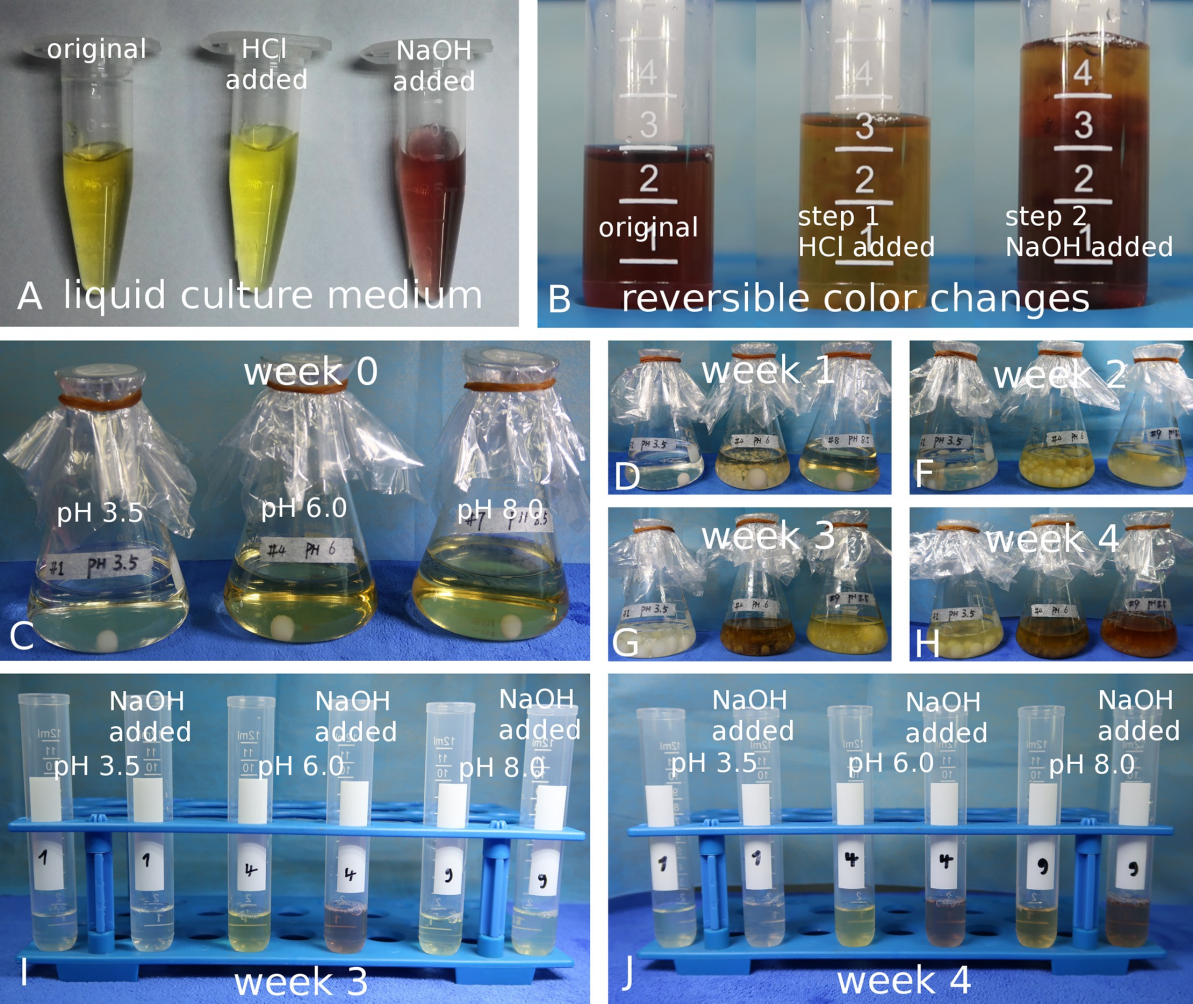
*T*. *rubrum* liquid cultures. A: Color change in 800 μl liquid media of strain STRB008. The liquid culture is originally yellow (left tube). After adding 100 μl 10% HCl the medium clears up (middle). By adding 100 μl 1M NaOH the liquid medium turns wine-red (right). B: The color change can be reversed and re-reversed by adding alternately acid or alkaline. The original liquid medium of strains STRB012 is wine-red (left tube). After adding 0.5 ml of 10% HCl the color changes to yellow (middle). By adding 1.5 ml 1M KOH the color change can be reversed to wine-red (right). C-H: Liquid cultures of strain STRB012 over a period of 4 weeks. The pH was adjusted to 3.5 (left flask), 6 (middle) or 8 (right). C: One day after inoculation the culture liquid is transparent with a slight yellow coloration. D: One week after inoculation the pH 6 trial has the highest growth rate, followed by the one of pH 8. Only minor changes of the culture liquid are visible. F: After two weeks of inoculation a yellow coloration had develops in the pH 6 trial. G: After 3 weeks the liquid of the pH 3.5 trial is transparent, reddish in the pH 6 and yellowish at the pH 8 trial. After 4 weeks the culture liquid of the pH 6 and 8 trial is reddish. I, J: Comparison of the culture liquid of strain STRB012 with culture liquid after adding 200μl 0.2M NaOH. I: Comparison of culture liquid 3 weeks after inoculation. From left to right: pH 3.5 trial, pH 3.5 with NaOH, pH 6 trial, pH 6 trial with NaOH, pH 8 trial and pH 8 trial with NaOH. An obvious color change to red is seen only in the pH 6 trial. J: Comparison 4 weeks after inoculation. A weak color change is observed in the pH 3.5 trial and an obvious change is seen in the pH 6 and 8 trial.

We also wanted to know whether the constitution of the color is directly influenced by the pH. For this, we cultivated strain STRB008 in liquid Tr1 medium for 3 weeks. We aliquoted 1ml of each culture liquid and added 100μl 10% HCl respectively 1M NaOH. The original liquid had a yellow color with a faint brown shading. After adding the acid, the liquid turned immediately into a sheer yellow, whereas after adding the alkaline, the liquid turned to wine-red (Fig 2A). The color change could be repeatedly reversed by adding acid or alkaline (Fig 2B and S1 Video). However, adding H_2_O_2_ bleached the liquid and no further color change could be induced.

### pH changes of the medium caused by *T*. *rubrum*

**Fig 3:**
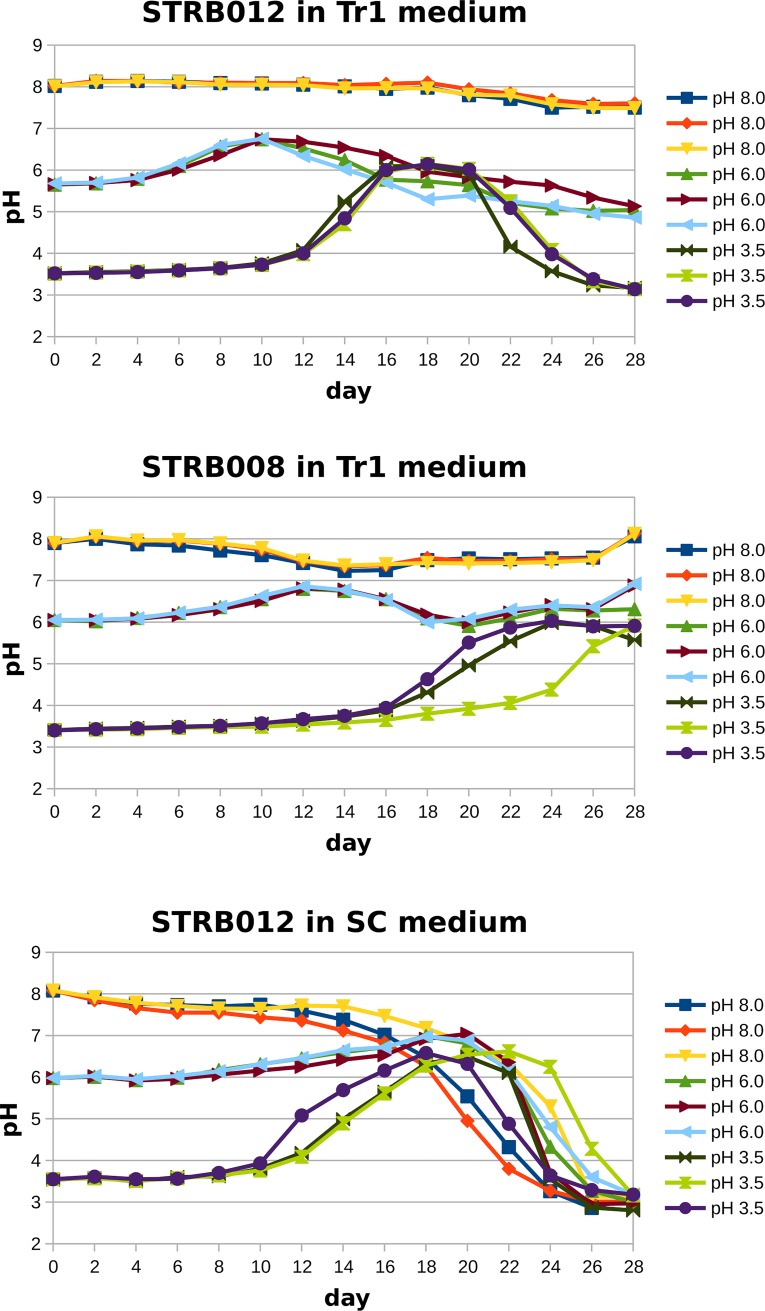
Change of the pH induced by *T*. *rubrum* observed over an period of 4 weeks. Before inoculation of the fungus, the pH was adjusted to 3.5, 6 or 8 and the pH was measured every second day. Each combination of fungal strain and pH has been performed in triplicate. A: *T*. *rubrum* strain STRB012 in Tr1 liquid medium. B: *T*. *rubrum* strain STRB008 in Tr1 liquid medium. C: *T*. *rubrum* strain STRB012 in SC medium.

To evaluate the influence of *T*. *rubrum* on the pH, we inoculated strain STRB008 and STRB012 to 150ml liquid Tr1 medium with a pH adjusted to 3.5, 6 and 8 (Fig 2C), each combination was set up three times. We measured the pH every second day, over a period of four weeks (Fig 2D–2G), by retrieving 2 ml liquid (Fig 2I and 2J). We detected a considerable change of the pH with a maximum amplitude of more than 2.5 (Fig 3A and 3B). In the low pH trial, the pH increased from approximately 3.5 to 6 and dropped afterwards again. In the pH 6 trial, the amplitude decreased, by approximately 0.8. At alkaline conditions, the pH increased at the beginning only slightly and afterwards dropped by approximately 0.5.

Every week, we visually classified the growth of the fungus and the color of the medium. Further, we added 1M NaOH to the aliquot, to trace whether color changes could be induced. We observed that the growth rate at pH 6 was the highest, followed by the trials with pH 8 and pH 3.5. At the beginning of the experiment and after one week, the culture liquids were merely clean with a trace of coloring due to the autoclaving. The first obvious color changes of the media were observed three weeks after inoculation in the pH 6 and 8 trials. Strain STRB012 turned, at pH 6, to sheer yellow and in one Erlenmeyer flask to brownish red. By adding 1M NaOH a color change to red could be induced (Fig 2I). After 4 weeks, all liquid media, at pH 6 and 8, had turned red (Fig 2J). Strain STRB008 turned the liquid media at pH 6 and as well at pH 8 into brownish red and after four weeks in all trials at pH 6 and 8 the liquid media had a dark color. At pH 3.5 a faint red color was visible by adding 1M NaOH to the media

### pH changes of the SC medium

We demonstrated, that all tested *T*. *rubrum* strains developed a strong red color on solid Tr1 medium, which had been adjusted to pH 6 and 8. On SC this color expression was missing. Since the color expression is strongly dependent on the pH, we wondered how the fungal growth influenced the pH of SC medium. We inoculated strain STRB012 to liquid SC medium with a pH adjusted to 3.5, 6 and 8. The course of pH alteration differed from the Tr1 medium (Fig 3C). About three weeks after inoculation, the pH started to drop and reached acid conditions of approximately pH 3 in the fourth week. An acidification happened in the pH 3.5, 6 as well as in the pH 8 trials.

## Discussion

The pigmentation of *T*. *rubrum* is variable and is influenced by many factors. We demonstrate, that the pigmentation is strongly influenced by the pH. Since the phenotype is quite constant among the fungal strains at constant conditions, but variable among the different conditions, we conclude that the environment is the determining factor in our experiments. At alkaline conditions, *T*. *rubrum* has a red pigmentation, whereas at acid condition *T*. *rubrum* has a yellow pigmentation.

**Fig 4:**
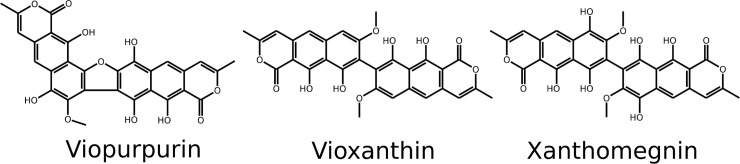
Chemical structures of Xanthomegnin (PubChem CID: 3032411), Viopurpurin (#73759973) and Vioxanthin (#119072), the three main pigments of *T*. *rubrum*. These compounds give *T*. *rubrum* the characteristic red pigmentation. They are belong to the family of 1,4-naphthoquinone and are synthesized from polyketides. The structural data of Xanthomegnin (PubChem CID: 3032411), Viopurpurin (#73759973) and Vioxanthin (#119072) were downloaded from NCBI PubChem database as xml file. Afterwards the files were converted with the Linux Babel software to pdb files, converted with Gnome GChemPaint software to a svg vector graphic and arranged with GNU program Inkscape.

*T*. *rubrum* possess three main pigments. [7] isolated and crystallized the three pigments from the mycelium and described them as Red Needles, Orange Plates and Purple Needles. Then these chemical components were isolated in greater quantity [8] and the structures of the substances were identified (Fig 4). The Orange Plates was named Xanthomegnin [9], the Purple Needle as Viopurpurin and the Red Needles as Vioxanthin [10] (Table 3). All three substances belong to the family of 1,4-naphthoquinone and are synthesized from polyketides [11]. Since Xanthomegnin is the main pigment in *T*. *rubrum* and isolated Xanthomegnin also changes color from yellow to red by alkalization, we attribute our observations of the color change mainly to Xanthomegnin.

**Table 3:** Comparison of the three main pigments of *T*. *rubrum*.

	Viopurpurin	Vioxanthin	Xanthomegnin
crystallized	purple*	red*	orange**
1N NaOH	Dark blue***	Purple***	Purplish-red***
Glac. Acetic Acid	Wine Red***	Yellow***	Yellow***

*[10]

**[8]

***[7]

The three pigments are typical for *Trichophyton* species and were detected only in very few other fungal species outside of the *Arthrodermataceae* family. Only few *Aspergillus* [12][13] and *Penicillium* [14] species are also known to synthesis these compounds. At least from Xanthomegnin and Vioxanthin it is known, that they are bioactive on the human body or even toxic. Xanthomegnin can influence the immune-response by inhibition of Inducible Nitric Oxide Synthase [15]. Further it has effects on mitochondria, as measured in rat liver mitochondria [16][17] and interacts with serum albumin [18]. Vioxanthin is a strong inhibitor of the human KLK5 and KLK7 genes. Both enzymes, Kallikrein-5 and Kallikrein-7, are most abundantly expressed in human skin and have an important role in skin desquamation [19]. Further, Semivioxanthin influences the TNF-a production through NF-k B and MAPK signaling pathways [20].

We demonstrate that *T*. *rubrum* changes the pH of the environment. [21] also observed a pH shift of a non-buffered liquid medium, with keratin as only source of nutrients, from 5 to approximately 8.5 within 96 hours after inoculation with *T*. *rubrum* conidia. [22] conclude that for *T*. *rubrum* an adequate pH is important for virulence. Whereas the human skin is mildly acidic, the highest proteolytic [23] and keratinolytic [24] activity in *Trichophyton* were measured at slight alkaline conditions in the range pH 7 to 8. Another important factor is the pH in the phagosome of macrophage. The intra-phagosomal pH in macrophages progressively decreases over 15 to 60 min down to pH 4 to 5 and this acidification is important for killing of diverse pathogens [25]. Sensing the intra- and extracellular pH and an adequate response is important for a cell. In fungi the PacC/Pal is a conserved pathway and regulates pH-conditioned gene expression. *T*. *rubrum* posses the transcription factor PacC and six pal genes [26]. The PacC/Pal pathway is well studied in *Aspergillus* species and [22] complied these data with the data of *T*. *rubrum*, and concluded that the signaling cascade is highly conserved in dermatophytes. It is known from other pathogenic fungi, animal and as well plant pathogenic fungi, that the pH regulation is a key factor for virulence, for example for *Botrytis cinerea* [27], *Candida albicans* [28] and *Colletotrichum gloeosporioides* [29].

One of the main pH-influencing factors in fungi is the release of urea or ammonia into the environment [28]. Therefore the α-amino group of an amino acid gets transferred by a transaminase to an α-ketoacid to form glutamate. Glutamate is than converted into ammonia and oxaloacetate by a glutamate dehydrogenase. Interestingly in *Saccharomyces cerevisiae* the glutamate dehydrogenase GDH2 is very sophisticated regulated, by 6 transcriptional regulation elements. Two of the elements behave as upstream activation sites, while the remaining four elements inhibit the effects of the two sites [30]. But at least for *Candida albicans* it is known that it can increase the pH of the environment by a second mechanism. It can rapidly neutralize acidic environments when utilizing carboxylic acids like pyruvate, α-ketoglutarate or lactate as the primary carbon source. Unlike cells growing in an amino acid-rich medium, this does not result in ammonia release and is genetically distinct [31]. Since both of the media we used in our experiments are rich in amino acid, we attribute the alkalization of the medium to ammonia activity. The main difference of the SC medium to the Tr1 medium is the high amount of ammonium sulfate, which leads to an increased acidification of the medium which leads to yellow pigmentation.

We conclude that the color of the pigmentation is influenced by pH. At high pH the pigmentation is red and at low pH yellow. We attribute both colors to the same chemical compound Xanthomegnin. We demonstrate that *T*. *rubrum* change the pH of cultivation media. One medium change to alkaline condition, another medium to acid condition. On the first medium *T*. *rubrum* has a wine-red pigmentation, on the other a yellow pigmentation.

## Supporting information

S1 Video Color change of liquid cultivation medium induced by HCl and NaOH. By adding HCl the medium changes to yellow, by adding NaOH it changes to wine-red.
(MP4)

## References

[1] Saunte DML, Piraccini BM, Sergeev AY, Prohić A, Sigurgeirsson B, Rodríguez‐Cerdeira C et al A survey among dermatologists: diagnostics of superficial fungal infections–what is used and what is needed to initiate therapy and assess efficacy? J Eur Acad Dermatol Venereol. 2019 33:421–427. 10.1111/jdv.15361 30468532

[2] Ghannoum M, Mukherjee P, Isham N, Markinson B, Del Rosso J, Leal L. Examining the importance of laboratory and diagnostic testing when treating and diagnosing onychomycosis. Int J Dermatol. 2018 57:131–138. 10.1111/ijd.13690 28653769

[3] Kidd S, Halliday C, Alexiou H, Ellis D. Descriptions of medical fungi. 2016 Third edition. https://mycology.adelaide.edu.au/.

[4] Baxter M. The stimulation of pigment production by *Trichophyton rubrum* on a new medium, Sabouraudia: Journal of Medical and Veterinary Mycology, 1964 3: 72–80. 5875372

[5] Guoling Y, Xiaohong Y, Lin J, Jin L, An L. A Study on Stability of Phenotype and Genotype of *Trichophyton rubrum*. Mycopathologia. 2006 161:205–12. 10.1007/s11046-005-0226-8 16552482

[6] Zhan P, Dukik K, Li D, Sun J, Stielow JB, Gerrits van den Ende B et al Phylogeny of dermatophytes with genomic character evaluation of clinically distinct *Trichophyton rubrum* and *T. violaceum*. Stud Mycol. 2018 89: 153–175. 10.1016/j.simyco.2018.02.004 29910521 PMC6002342

[7] Wirth JC, O'Brian PJ, Schmitt FL, Sohler A. The isolation in crystalline form of some of the pigments of *Trichophyton rubrum*. J Invest Dermatol. 1957 29: 47–53. 10.1038/jid.1957.71 13475944

[8] Blank F, Day WC, Just G. Metabolites of pathogenic fungi. II. The isolation of xanthomegnin from *Trichophyton megnini* Blanchard 1896. J Invest Dermatol. 1963 40: 133–7. 13971503

[9] Wirth JC, Beesley TE, Anand SR. The isolation of xanthomegnin from several strains of the dermatophyte, *Trichophyton rubrum*. Phytochemistry 1965 4: 505–509.

[10] Blank F, Ng AS, Just G. Metabolites of pathogenic fungi: V. Isolation and tentative structures of vioxanthin and viopurpurin, two colored metabolites of *Trichophyton violaceum*. Canadian Journal of Chemistry 1966 44: 2873–2879.

[11] Fürtges L, Obermaier S, Thiele W, Foegen S, Müller M. Diversity in Fungal Intermolecular Phenol Coupling of Polyketides: Regioselective Laccase-Based Systems. Chembiochem. 2019 20: 1928–1932. 10.1002/cbic.201900041 30868712

[12] Robbers JE, Hong S, Tuite J, Carlton WW. Production of xanthomegnin and viomellein by species of *Aspergillus* correlated with mycotoxicosis produced in mice. Appl Environ Microbiol. 1978 36: 819–23. 736540 10.1128/aem.36.6.819-823.1978 PMC243152

[13] Kamiya K, Arai M, Setiawan A, Kobayashi M. Anti-dormant Mycobacterial Activity of Viomellein and Xanthomegnin, Naphthoquinone Dimers Produced by Marine- derived *Aspergillus* sp. Nat Prod Commun. 2017 12: 579–581. 30520600

[14] Stack ME, Eppley RM, Dreifuss PA, Pohland AE. Isolation and identification of xanthomegnin, viomellein, rubrosulphin, and viopurpurin as metabolites of *Penicillium viridicatum*. Appl Environ Microbiol. 1977 33: 351–5. 848956 10.1128/aem.33.2.351-355.1977 PMC170690

[15] Alvi KA, Baker DD, Stienecker V, Hosken M, Nair BG. Identification of inhibitors of inducible nitric oxide synthase from microbial extract. The Journal of Antibiotics 2000 53: 496–501. 10908113

[16] Kawai K, Akita T, Nishibe S, Nozawa Y, Ogihara Y, Ito Y. Biochemical studies of pigments from a pathogenic fungus *Microsporum cookei*. III. Comparison of the effects of xanthomegnin and O-methylxanthomegnin on the oxidative phosphorylation of rat liver mitochondria. J Biochem. 1976 79: 145–52. 10.1093/oxfordjournals.jbchem.a131041 939756

[17] Ito Y, Kawai K, Nozawa Y. Biochemical studies of pigments from the pathogenic fungus, *Microsporum cookei*. J Biochem. 1973 74: 805–10. 10.1093/oxfordjournals.jbchem.a130306 4271697

[18] Kawai K, Cowger ML. 1982 Spectrophotometric study of the interaction of xanthomegnin with serum albumin. Res Commun Chem Pathol Pharmacol 35: 499–513. 7079576

[19] Teixeira TS, Freitas RF, Abrahão O Jr, Devienne KF, de Souza LR, Blaber SI et al Biological evaluation and docking studies of natural isocoumarins as inhibitors for human kallikrein 5 and 7. Bioorg Med Chem Lett. 2011 20: 6112–5. 10.1016/j.bmcl.2011.08.044 21903387

[20] Yang XY, Cai SX, Zhang WJ, Tang XL, Shin HY, Lee JY et al Semi-vioxanthin isolated from marine-derived fungus regulates tumor necrosis factor-alpha, cluster of differentiation (CD) 80, CD86, and major histocompatibility complex class II expression in RAW264.7 cells via nuclear factor-kappaB and mitogen-activated protein kinase signaling pathways. Biol Pharm Bull. 2008 12:2228–33. 10.1248/bpb.31.2228 19043204

[21] Silveira HC, Gras DE, Cazzaniga RA, Sanches PR, Rossi A, Martinez-Rossi NM. Transcriptional profiling reveals genes in the human pathogen *Trichophyton rubrum* that are expressed in response to pH signaling. Microb Pathog. 2010 48: 91–6. 10.1016/j.micpath.2009.10.006 19874884

[22] Martinez-Rossi NM, Peres NT, Rossi A. Pathogenesis of Dermatophytosis: Sensing the Host Tissue. Mycopathologia. 2017 182: 215–227. 10.1007/s11046-016-0057-9 27590362

[23] Asahi M, Lindquist R, Fukuyama K, Apodaca G, Epstein WL, McKerrow JH. Purification and characterization of major extracellular proteinases from *Trichophyton rubrum*. Biochem J. 1985 232: 139–144. 10.1042/bj2320139 3910025 PMC1152850

[24] Sharma A, Chandra S, Sharma M. Difference in keratinase activity of dermatophytes at different environmental conditions is an attribute of adaptation to parasitism. Mycoses. 2012 55:410–5. 10.1111/j.1439-0507.2011.02133.x 22032519

[25] Nüsse O. Biochemistry of the Phagosome: The Challenge to Study a Transient Organelle. ScientificWorldJournal. 2011 11: 2364–2381. 10.1100/2011/741046 22194668 PMC3236389

[26] Martinez-Rossi NM, Persinoti GF, Peres NT, Rossi A. Role of pH in the pathogenesis of dermatophytoses. Mycoses. 2012 55: 381–7. 10.1111/j.1439-0507.2011.02162.x 22211778

[27] Rascle C, Dieryckx C, Dupuy JW, Muszkieta L, Souibgui E, Droux M et al The pH regulator PacC: a host-dependent virulence factor in *Botrytis cinerea*. Environmental Microbiology Reports 2018 10: 555–568. 10.1111/1758-2229.12663 30066486

[28] Vylkova S. Environmental pH modulation by pathogenic fungi as a strategy to conquer the host. PLoS Pathog 2017 13: e1006149 10.1371/journal.ppat.1006149 28231317 PMC5322887

[29] Miyara I, Shafran H, Davidzon M, Sherman A, Prusky D. pH Regulation of ammonia secretion by *Colletotrichum gloeosporioides* and its effect on appressorium formation and pathogenicity. Mol Plant Microbe Interact. 2010 23: 304–16. 10.1094/MPMI-23-3-0304 20121452

[30] Miller SM, Magasanik B. Role of the complex upstream region of the GDH2 gene in nitrogen regulation of the NAD-linked glutamate dehydrogenase in *Saccharomyces cerevisiae*. Mol Cell Biol 1991 11:6229–47. 10.1128/mcb.11.12.6229 1682801 PMC361811

[31] Danhof HA, Vylkova S, Vesely EM, Ford AE, Gonzalez-Garay M, Lorenz MC. Robust Extracellular pH Modulation by *Candida albicans* during Growth in Carboxylic Acids. MBio. 2016 7: 01646–16. 10.1128/mBio.01646-16 PMC5111404 27935835

